# Feminism and literary translation: A systematic review

**DOI:** 10.1016/j.heliyon.2022.e09082

**Published:** 2022-03-16

**Authors:** Isra Irshad, Musarat Yasmin

**Affiliations:** Department of English, University of Gujrat, Hafiz Hayat Campus, Gujrat, Pakistan

**Keywords:** Feminism, Feminist translation studies, Translated novels, Review

## Abstract

Feminist translation theory demeans the culture of the patriarchal hegemony of translation. The purpose of this study is twofold: to investigate the main trends in the studies on feminism and literary translation and to analyse the main ways through which feminist translation theory has been applied by various researchers in the studies of translated novels. To this end, the databases of EBSCO, ProQuest, Taylor and Francis, ETHOS, and Google Scholar have been explored, and thirty-three studies published between 2005 and 2021 have been analysed. A systematic review was used as a research methodology, and the studies were analysed using a content analysis method. The findings revealed that there are very few significant studies on feminism and translation of novels, and at least until the sharp increase in interest in research in the field emerged in 2019. Moreover, other studies have concluded that feminist translation theory has focused on examining the impact of gender consciousness and translator ideology on the translation process, exploring feminist translation strategies, and analysing the transmission of gendered language in the translated text. The findings have provided feminist and translation studies researchers with a comprehensive understanding of the current state of applied feminist translation theory in the studies addressing translated novels.

## 1 Introduction

The translation is not only a process of transferring linguistic codes from one language to another, but it has also become a political activity (Li, 2020). After the 1990s, scholars started analysing it from the perspective of cultural theories (Hou et al., 2020). In this context, the linguistic theories applied to translation are critiqued, as they “have moved from word to text as a unit, but not beyond” (Bassnett, and Lefevere, 1990: 4), and these theories do not take into account the text in its cultural environment. Snell Hornby (1990) introduced the term “cultural turn” to highlight the cultural aspect of translation. Translating is now regarded as a process of rewriting which involves the issues of ideology, power, and manipulation (Lefevere, 1992; Levy, 2000). The issue of ideological impact on translations was not fully recognized in the past (Baumgarten, 2012). Against this backdrop, the feminist theory came to be applied to translation.

Feminist theory aims at understanding the nature of gender inequality (Shuo and Min, 2017); by analysing women's life experience and their social roles. It uses various labels to define feminist trends which indicate the social, economic, and historical contexts in which they emerged: French feminism, Enlightenment feminism, liberal feminism, black feminism and so on (Escudero-Alías, 2021). It seeks to challenge methodologies, traditions, and priorities in all spheres of life and demands equal rights for women (Flotow, 1997). It indicates “a world view that values women and that confronts systematic injustices based on gender” (Chinn and Wheeler, 1985: 74). It has emerged from feminist movements of the nineteenth and twentieth centuries. The movements announced “a widespread call for a major reassessment of the concepts, theories, and method employed within and across the academic disciplines” (Hesse-Bibe et al., 2004: 57). These movements highlighted the significance of language in constructing womanhood and gender (Ergu, 2013), and thus, in maintaining social inequalities (Simon, 1996).

Feminism attempts to (re)claim language to deconstruct patriarchy. In this context, in the 1970s & 1980s, an alternative woman's language was created taht dismantled patriarch language, and made women linguistically visible. Flotow observed unconventional spellings, subverted semantic and grammatical systems, puns and neologism (as cited in Flotow, 1991). Translating these writings requires some resistant translation strategies which laid the ground for feminist translation studies (FTS). The focus of FTS is to include feminist ideology in translation. Feminist translators Godard, and Harwood have an ideological motivation and believed that language and translation are not neutral, “innocent” acts (Simon, 1996), and are significant tools for legitimizing or subverting the status quo (Castro, 2013).

The feminist translation developed in Canada in the multilingual situation having the purpose of subverting the culture of patriarchal hegemony of translation. Canadian feminist translations were seen as the “method of translating the focus on and the critique of patriarchal language by feminist writers in Quebec (Brevet, 2019:11). Feminist Translation Studies identifies and critiques “the tangle of concepts which relegate both women and translation to the bottom of the social and literary ladder” (Simon, 1996, p.1). It challenges the notion of fidelity present in Translation Studies. It posits that feminist translators have the right to intervene in the source text to make women visible, where fidelity is seen towards the writing project in which the writers and translators participate, and not necessarily towards the writers or readers (Simon, 1996). Moreover, feminist translation focused on the translator's subjectivity and made women's voice heard in the world (Lotbiniere-Harwood, S., 1989), and the main contributors in feminist translation studies were Flotow (1991), Simon (1996), and Godard (1984). Castro (2009) stated that “Canadian feminist translation (…) is a school of work and thought that defends the incorporation of the feminist ideology into translation because of the need to establish new ways of expression that make it possible to free language and society from their patriarchal burden”.

Feminist translation has an interventionist approach to translation (Flotow, 1991), and intends to “womanhandle” the text (Godard, 1989). The practices of feminist translation involve translating women's work, feminist works, and challenging patriarchal translation of the women's texts (Simon, 1996). The feminist translator uses several feminist translation strategies, such as hijacking, supplementing, prefacing, and footnoting as discussed by Flotow (1991). In supplementing, “the ST (source text) is supplemented by its translation, matured, developed, and given an afterlife” (Flotow, 1991). The Feminist translator also uses prefaces and footnotes to indicate their presence. Godard's example of prefacing of Brossard's feminist text is commonly presented as an example. Hijacking is another feminist translation strategy in which the translator makes extreme interferences in the translation process by hijacking the Source text (Flotow, 1991).

The Canadian school of feminist translation is usually associated with the first wave of feminist translation (Le Bervet, n.d). From the 1990s onwards, the research in feminist translation has moved from the Canadian school to issues based on transnational feminist translation. It finds its alignment with the second wave of feminism in terms of ideas for example, diversity, intersectionality, and inclusivity. Castro (2009) claims that this approach is identified as the second wave of feminist translation which is different from the Canadian school which is identified as a universal paradigm. She says that though the Canadian school has contributed to the TS, there is a need to redefine the aim of feminist translation. The same idea was proposed in 1997 by Massardior- Kenney. The problem comes with the assured stable definition of woman and feminism. There is a clear necessity to acknowledge and accept how complex these terms are. She claims that there is a need to translate women's writing where gender is not explicit and it entangles with other issues. Massardier Kenney (1997) says that a redefinition of feminist translation will “contribute to an examination of the translating activity in general; by emphasizing the importance of gender categories and the mechanisms through which the “feminine” is excluded or is valued” and, to show that “translation is a crucial form of cultural production” (1997, p.66). Starting in 2000, we see that work on feminist translation studies are built on this redefinition by re-evaluating historical texts and their translations, e.g. Wolf (2005) has investigated the works of two 18^th^ century feminist translators (Gottshed and Huber). Moreover, she has also evaluated publishing house guidelines on non-sexist language. Castro and Ergan also have investigated Feminist Translation in minority languages (Castro, 2013). New areas of research are being explored, e.g. criticism of phallocentric translation of feminist work (Bogic, 2011), investigation of para-translation of feminist work (Castro, 2009). Rosario (2005) posits that post-structuralist translators have shifted toward the more inclusive approach due to the evolution of feminism. The Canadian school of Feminist Translation is not suitable to deal with a plurality of identities. Now the approaches like queer, lesbian, and gay translation have emerged as tools to resist heteronormativity.

This theory of feminist translation has been used to analyse several literary genres, but this present research only takes into account those studies, which have analysed the translated novels only. Furthermore, by applying this theory, various studies have been conducted in different cultural contexts around the world. For example, in the Chinese context, Tang (2018), by using the feminist perspective, has analysed gender issues in the Chinese translations of Chinese American women's literature. Furthermore, in the Iranian context, Mohammadi (2014) has investigated the role of gender ideology in the Persian translations of *Mrs. Dalloway.* In the same way, in the American context, Modrea (2005) has investigated the extent to which the translated text is reconstructed as feminist.

The above-mentioned studies indicate that feminist translation theory has been used in various cultural and historical contexts. Moreover, in these studies, various perspectives have been considered: Translation strategies used by feminist translators - Chen and Chen (2016); Shuo and Min (2017); Qiu (2019); Hou et al. (2020); Abdel and Allam (2018); the impact of gender ideology in the process of translation - Modrea (2005); Baya (2019); Mohammadi (2014), and so on. The field lacks a systematic literature review of research on feminist theory applied to novel translation. There is a need of conducting a review of research falling within this paradigm as it will help the feminist theory and translation studies researchers to develop a comprehensive understanding of the current state of application of feminist translation theory in the literary genre of novels by critically evaluating the existing literature. The present study thus fills this gap by reviewing the relevant research and by highlighting the gap for future research in this paradigm. The present study has attempted to answer the following research questions:

1) What are the recent trends in the research on feminism and the translation of novels?
2) What are the ways through which feminist translation theory has been applied in the studies addressing translated novels?

## 2 Method

A systematic review method is employed in the present study, based on research questions that govern which studies to be included for review (Uman, 2011). The present study intends to investigate the trends in the research on feminism and translation of novels, and to analyse the ways through which feminist translation theory was applied in such translation. Moreover, there is a difference between literature review and systematic review. A systematic review is thoroughly well organized (Kowalczyk and Truluck, 2013), and the studies are methodically selected from available studies in particular databases. On the other hand, the literature review method is less systematic.

### 2.1 Data collection and analysis

The research articles, conference papers and dissertations that were relevant to the research questions and aims of this research were collected from EBSCO, ProQuest, Taylor and Francis, Ethos and Google Scholar, written in English and published during 2005–2021. The present study investigated the trends and ways feminist translation theory was applied in the included studies over the last sixteen years. The selection of search terms, “feminist translation and novel”, “feminism, translation, novel”, “Feminist identities and translation novel”, was guided by research questions.

Feminist translation is “a method of translating the focus on and critique of "patriarchal language" by feminist writers” (Flotow, 1991). Feminist identity in translation explains how a female image is translated in the translated texts. These terms of “feminist translations” and “feminist identity in translation” were searched along with the word “novel” to collect the studies on feminist translations or feminist identities in the translated novels. From the corpus of analysis, the researchers included only relevant research works for analysis.

The included studies were analysed by performing content analysis. It allowed researchers to compare, contrast and categorize the data (Fraenkel and Wallen, 2000). The two research questions were divided into various subcategories. The first research question was based on descriptive information. On the other hand, the second research question demanded a detailed reading of the included studies. Consider the following Table 1 in this regard:

**Table 1:** Subcategories within research questions.

Research questions	Subcategories
What are the current trends in feminism and novel translation research?	**By year distribution of studies:**
	The selected studies are categorized to determine how many studies are conducted each year.
	**Research methods used in reviewed studies:**
	The selected studies are categorized to determine the most commonly used research methods in the selected studies.
	**Languages involved in the ST (Source text) and TT (target text) of the novels in selected studies:**
	The reviewed studies would be analysed to determine the languages involved in the ST (Source text) and TT (target text) of the novels in the selected studies.
What are the main ways through which feminist translation theory has been applied in the selected studies?	**The analysis involves the main ways feminist translation theory has been applied in selected studies:**
	Examination of the impact of gender consciousness and ideology of the translator on the translation activity
	Exploration of the feminist translation strategies
	Analysis of the transmission of gendered language in the TT

**Figure 1:**
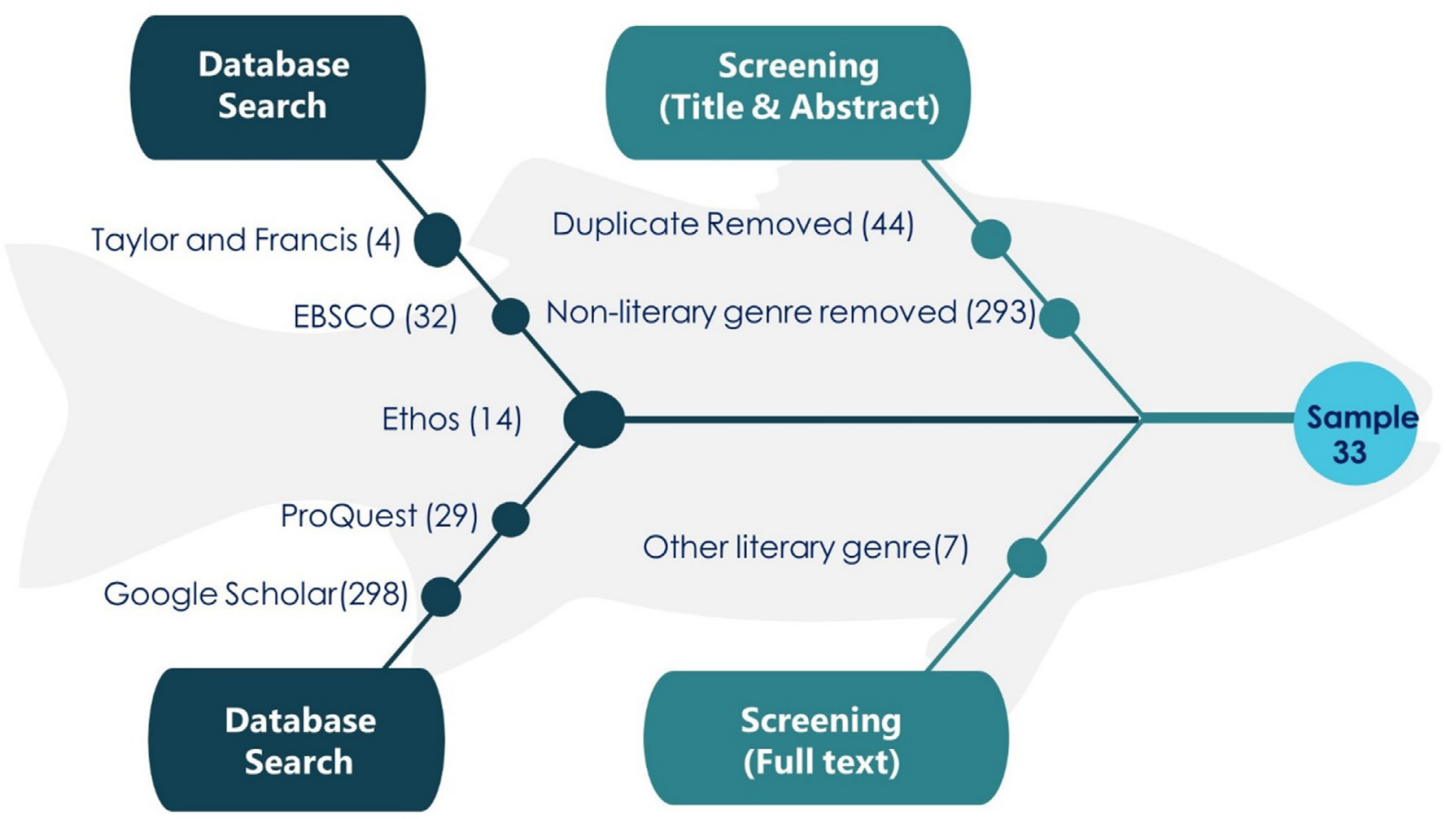
The selection process of the studies.

After inputting the search terms on EBSCO, ProQuest, Taylor and Francis, Ethos and Google Scholar, only those articles/theses which met the search criteria were included (i.e., those that were completely accessible, deal with feminism, and translated novels, and excluding studies that fall within the following criteria: books and chapters on feminist translation, research articles on feminist translation written in languages other than English; articles discussing feminist translation with theoretical perspectives, articles on feminist translations which are not accessible). Thus, initially the numbers of selected articles from the databases are 32 from EBSCO, 4 from Taylor and Francis, 29 from ProQuest, 298 from Google Scholar, and 14 from Ethos. After the removal of duplicated studies, the resulting studies are 333. The inclusion criteria of the studies included: a) research on translated novels from the feminist perspectives b) publications between 2005 and 2021 c) research article, thesis and conference paper. After examining the title and abstract, 293 studies were excluded as they deal with the non-literary genre. The rest of the forty studies were reviewed for eligibility. Among these studies, seven were removed as they were related to other literary genres (not novels) and with merely descriptive work. After the mentioned process, the thirty-three studies were reviewed in the present study. Figure 1 below (adapted from Liberati et al., 2009) is a useful tool to understand this result-filtering process:

## 3 Results

This section will provide readers with an overview of the results found on the trend in the research on feminism and translation of novels, and the extracted aspects regarding the application of the feminist translation theory.

### 3.1 Trends in the research on feminism and translation of novels

The subcategories in research question number one are by year distribution of the studies, research methods and the languages involved in the source text and target text of the selected novels. Each of these categories will be discussed in this section.

#### 3.1.1 By year distribution of the studies

**Figure 2:**
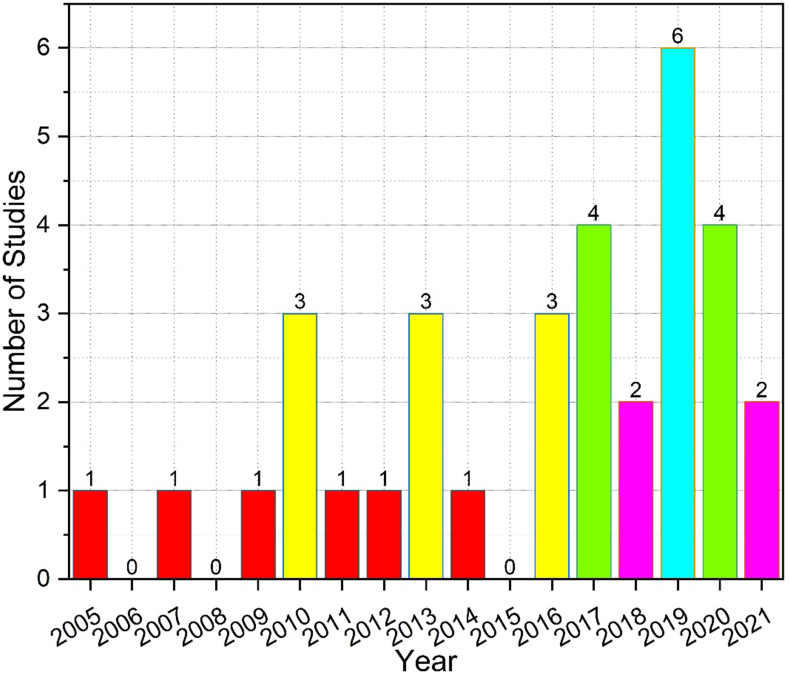
Distribution of included studies by publication year.

The distribution of the selected studies on translation of novels and feminism by year is shown in Figure 2.

Figure 2 illustrates the significant rise from 2019 onwards, in the number of interdisciplinary studies on feminist theory and translation of novels (see for example Mei (2019), Qing Qiu (2019), Li and Zhang (2019), and Baya (2019)). However, the number of studies remained constant in 2010, 2013, 2016, and 2017 (including two studies each year, see for example Paleczek (2010), and in 2005, 2007, 2009, 2011, 2014 (including one study each year, see for example Li (2007).

#### 3.1.2 Research methods used in the reviewed studies

In order to see the trends in the selected studies in our research, it is necessary to know the research methods used in these studies. Figure number three below will illustrate the research methods used in these studies.

**Figure 3:**
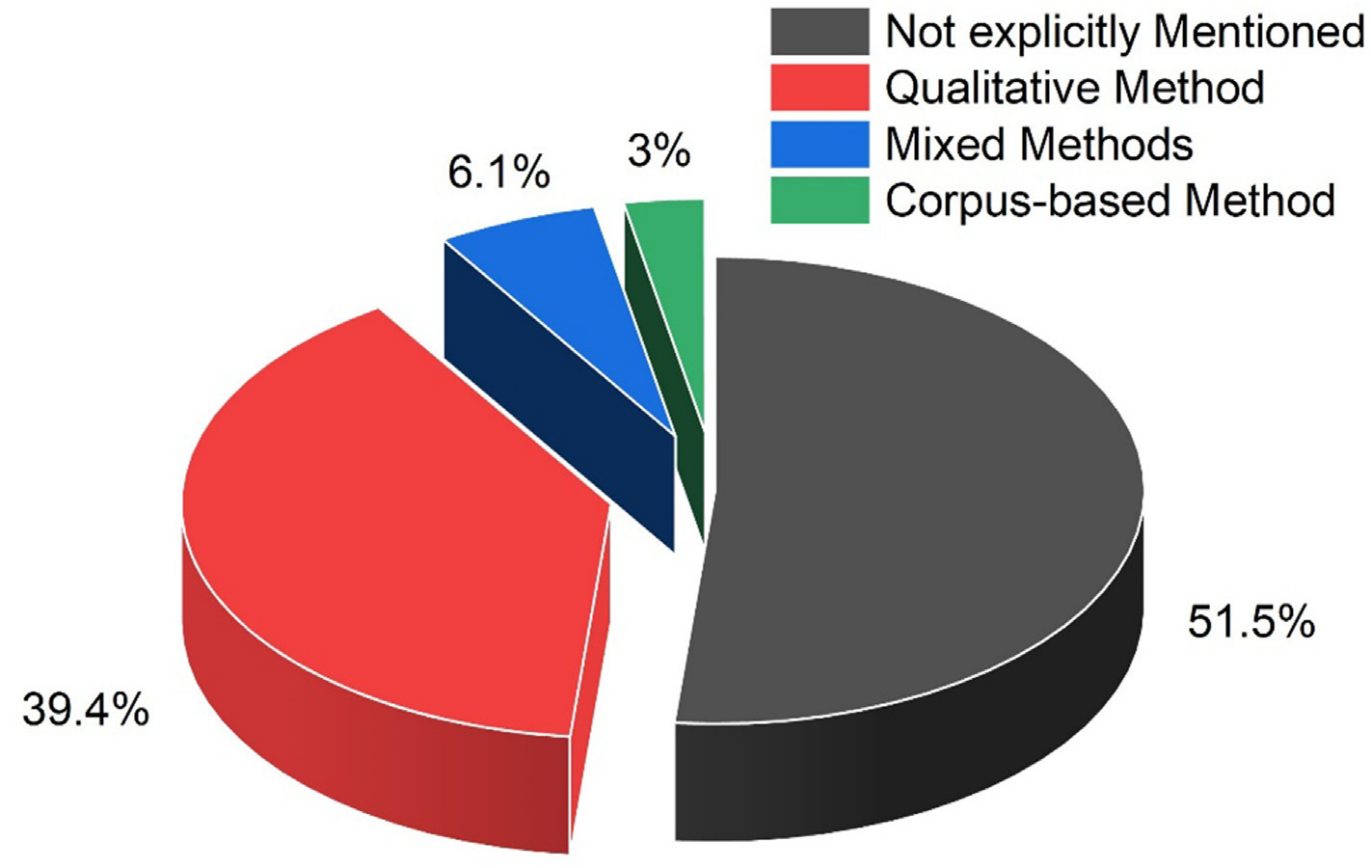
Research methods used in the reviewed studies.

Figure 3 highlights that seventeen studies have not explicitly mentioned the adopted research methods (see for example, Paleczek (2010), and Chen and Zhang (2016)); where thirteen have adopted qualitative research methods (see Modrea (2005); Hsing (2011) etc.) and only two studies have explicitly mentioned that they have conducted studies with the combination of qualitative and quantitative methods (see Mohammadi (2014)). Moreover, only one piece of research done by Bateni et al. (2013) has adopted the corpus-based method.

#### 3.1.3 Languages involved in the ST and TT of the novels in the selected studies

**Figure 4:**
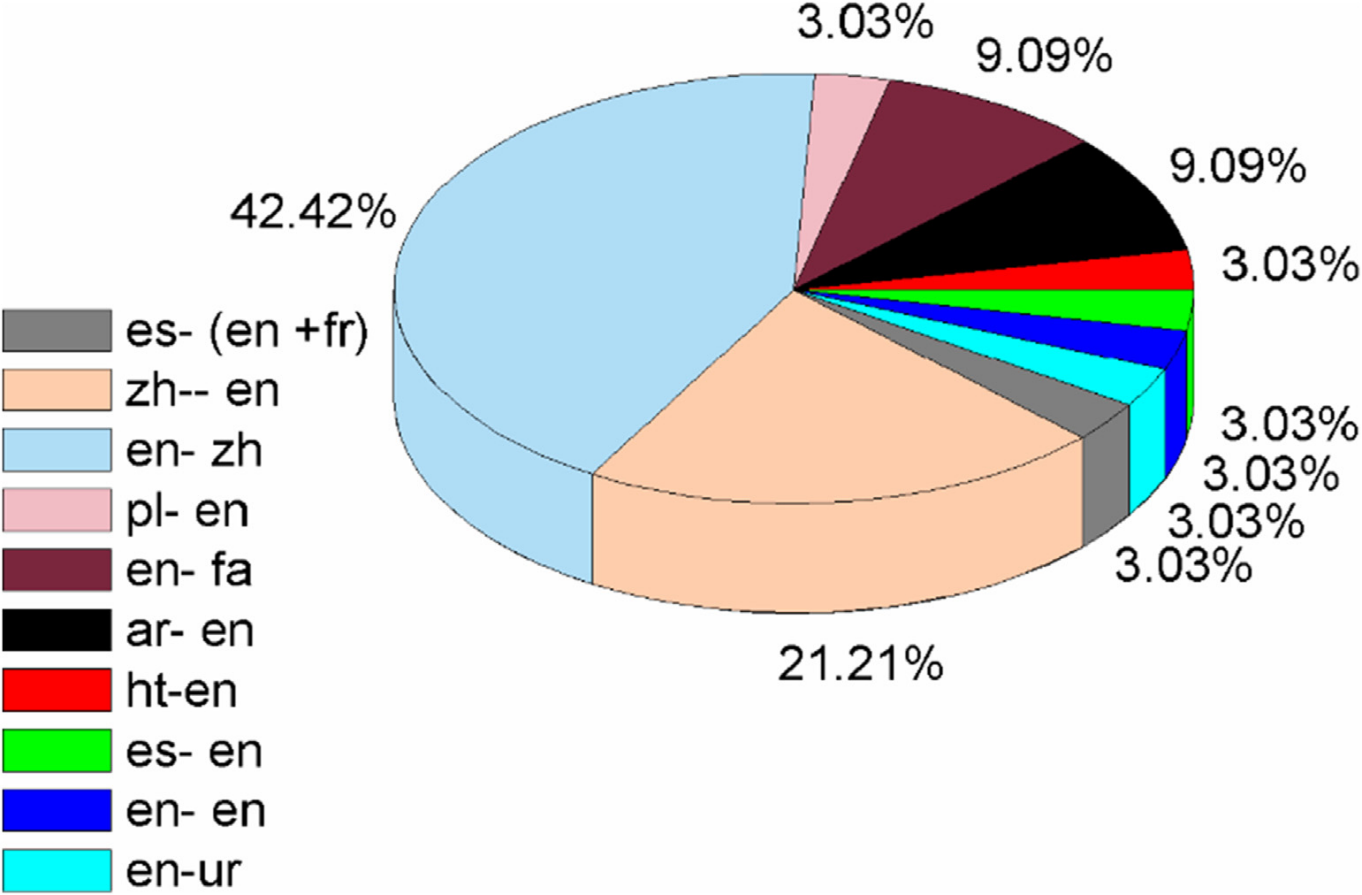
Languages involved in the ST and TT of the novels in the selected studies.

Figure 4 indicates the languages involved in the translation process from the source text to the target text of the selected novels. The language codes have been adopted from the ISO language code list. The blue portion (42.42%) covers a large portion of the figure, showing that most of the analysed translated novels were translated from English into Chinese (n = 14), see for example Hsing (2011), Chen and Zhang (2016), and so on. The frequencies of the languages used and examples of the works translated from one language to another are given as follows:

Chinese (zh) - English (en) = 7, (see for example Shou and Min (2017))

English (en) - Persian (fa) = 3, (see for example Moghaddas (2013))

Arabic (ar)- English (en) = 3, (see for example Allam (2018))

Spanish (es) - (English (en) + French (fr)) = 1, (see Modrea (2005)

Polish (pl) - English (en) = 1, (see Paleczek (2010))

Haitian (ht)-English (en) = 1, (see Mei (2019))

English (en) – Urdu (ur) = 1, (see Shaheen et al. (2021))

### 3.2 Application of feminist translation theory in the translation of novels

This section has dealt with the application of feminist translation theory in the studies addressing translated novels. It means to review which approaches or methods have been used by various researchers when they have used feminist translation theory in their respective studies. We have found three specific ways of applying feminist translation theory; i.e., 1) the impact of gender consciousness and ideology of the translator on the translation activity, 2) feminist translation strategies in the translated novels, and 3) transference of gendered language in the translated novels.” These are discussed in detail in the following section. Moreover, some miscellaneous findings of the research related to the literary translation of novels and feminism are also discussed.

## 4 Discussion

The current rising number of studies on translated novels from a feminist perspective might be linked to the rising popularity of Feminism, as a social and cultural phenomenon, all around the world, and particularly the rapid development of the interdisciplinary nature of translation studies in recent years. The intense interest in the research on this topic suggests the possibility of a fast increasing admiration in the up-coming years. The analysis of the reviewed studies in terms of languages of the translated works indicates that mostly the novels, which are translated from English (en) - Chinese (zh) (n = 14) have been analysed from the feminist perspectives. Their translations into Chinese in recent years can be attributed to the rapid rise of feminist movements in China, and with the emergence of China as one of the current global superpowers where much literature is being translated into the Chinese language. The following discussion is based on the ways the feminist translation theory has been used in the analysis of the translation of novels, and thus, this section presents the results for the RQ2. The Tables 2 and 3 present the main and miscellaneous findings respectively.

**Table 2:** The main findings of the studies applied feminist translation theory.

Author/s and year	Focus	Research Methodology	SL-TL	Novel/s, Author-Translators	Findings
Modrea (2005)	Translator’ ideology	Qualitative	Spanish- English + French	*Tres Tristes Tigres* by Guillermo Cabrera Infante – (translators: Bensoussan, Levine).	Bensoussan's translation is a straightforward, direct translation, with few modifications; Levine has clearly shown her voice in translation
Paleczek (2010)	Translation of grammatical gender	Not explicitly mentioned	Polish- English	*Dom dzienny, dom nocny* by Tokarczuk (translator: Antonia Lloyd).	Tokarczuk's play with language can be “rewritten” in English—though English lacks pervasive grammatical gender, it does not lack corresponding cultural and patriarchal constructions.
Hsing (2011)	Impact of gender consciousness in the translation	Qualitative	English-Chinese	Children's literature translated from British English to Chinese Alice's Adventures in Wonderland (1865), The Princess and the Goblin (1872), Treasure Island (1883), The Happy Prince and Other Tales (1888), The Wind in the Willows (1908), Peter Pan (1911).	Taiwanese Female translators have intervened in the Culture, and have constructed their gender identities. A large proportion of male translators in the selected texts share similarities and tend to use language that is stronger, more aggressive, and direct to interpret the source narrative.
Miletich (2012)	Translation of grammatical gender	Qualitative	Spanish- English	*Pronombres Personales* by Isaac Chocrón (translator: Miletich).	The existence of texts that contain words that can be masculine or feminine in Spanish provides an extra challenge when transferring the Spanish text into English, a language that does not seem to have such an abundance of these explicit terms.
Bateni et al. (2013)	Feminist translation strategies	Corpus-based research	English-Persian	*Tess D'Urbervilles* by Hardy (translators: Siravan Azad and Mina Sarabi); *Unaccustomed Earth* by Jhumpa Lahiri (translators: Amir Mehdi Haghighat and Goli Emami); *Mrs. Dalloway* by Virjinia Woolf (translators: Parviz Dariush and Farzaneh Taheri); *Pride and Prejudice* by Jane Austen (translators: Reza Rezaei and Sousan Ardekani); *Wuthering Heights* by Emily Bronte (translators: Kamran Parvaneh and Fatemeh Amini); *A Thousand Splendid Suns* by Khalid Husseini (translators: Mehdi Ghabraei and Somayyeh Ganji).	Female translators have used footnotes in their translations more than male translators.
Moghaddas (2013)	Gender differences in translation	Qualitative	English-Persian	Not mentioned explicitly	There is no significant difference between the Iranian male and female translators' translations in terms the translation accuracy.
Mohammadi (2014)	Translators' ideology	Qualitative + Quantitative	English- Persian	*Mrs. Dallowa*y (1925) by Virginia Woolf (translators: Darush and Taheri).	Almost all the manipulated selected words are used by the male translator. Statistical data, on the other hand, revealed a difference between positive and negative gender values in which the negative choices are employed more by the male and the positive ones more by the female translator
Chen and Zhang (2016)	Translators' subjectivity	Not explicitly mentioned	English-Chinese	*The Old Man and the Sea* by Ernest Hemmingway-(translator: Eilen Chang)	Feminist translators' subjectivity is manifested greatly in the translation practice of Eileen Chang
Chen and Chen (2016)	Feminist translation strategies	Not explicitly mentioned	English-Chinese	*The Old Man and the Sea* by Ernest Hemmingway (translator: Zhang Ailing).	The feminist translation strategies of prefaces, supplementing, and hijacking have been used.
Yuan (2016)	Gender consciousness in translated children's	Qualitative	English-Chinese	*The little Berry* by J.M Berry (translator: Liang).	Peter's gender (the character in children's literature) has been consistently disguised in the target text. The mystification of Peter Pan's gender is discussed in light of the conceptualization of childhood in China and the development of domestic children's literature and feminist movements in the 1920s, highlighting the role the target culture context plays in translation.
Shuo and Min (2017)	Feminist translation strategies	Not explicitly mentioned	English- Chinese	*The Old Man and the Sea* by Ernest Hemmingway; *The Legend of the* *Sleepy Hollow* by Washington Irving (translator: Zhang Ailing).	The feminist translation strategies of prefacing, supplementing, and hijacking have been used.
Shuo and Min (2017)	Feminist translation strategies	Not explicitly mentioned	Chinese-English	*The Golden Cangue by* Eileen Chang	The feminist translation strategies of prefaces, footnoting supplementing, hijacking have been used
Raidah (2017)	Impact of gender consciousness in the translation	Qualitative	Arabic-English	Corpus of 10 novels written by Arab women writers, translated by women translator.	The gender of authors and translators did not impact the way Arab women are represented
Allam (2018)	Feminist translation strategies	Qualitative	Arabic- English	*Professor Hanaa* by Reem Bassiouney (translator: Laila Helmy).	The TT is womanhandled by prefacing and footnoting, supplementing and hijacking to fit certain feminine politics by 7.2%, 75.3% and 17.5% lexically and semantically to exalt the main female figure and to demean the male figures.
Tang (2018)	Translator's ideologies and the impact of gender consciousness in	Qualitative	English -Chinese	*The Joy Luck Club* by Amy Tan (1989) (translators: Cheng Naishan, Yan Yingwei, and Peihua); *The Kitchen God's Wife* (1991) by Amy Tan (translators: Yang De, Ling Yue, and Yan Wei, Zhang Deming).	The hijacking and supplementing that female translators use in a few cases reflect that they have a better understanding of Tan's thoughts and intentions and attempt to convey them to the readers, which also shows their gender consciousness and attitudes toward women's awakening. In contrast, the male translators prefer rewriting and omission, with a result that their translations deviate from traditional translation ethics of fidelity
Qing Qiu (2019)	Impact of gender consciousness in the translation	Qualitative	English- Chinese	*To the Lighthouse* by Virginia Woolf; (translators: Ma Ainong, Qu Shijing).	The analysis reveals the differences between female translation and male ones as a result of their gender consciousness.
Li and Zhang (2019)	Linguistic choices of the translators	Not explicitly mentioned	English- Chinese	*Persuasion* by Jane Austen (translators: Sun Zhili and Qiu Yin).	Female preferences of exclamatory sentences and rhetorical questions, sentence-final particles, reduplicated words as well as prefaces and footnotes.
Baya (2019)	Translators' ideology	Not explicitly mentioned	Arabic- English	*Women at Zero* *Point* by Nawel Saadaouis (translator: Hatata); *Memory of the Flesh* by Ahlem Mosteghanemi (translator: Raphael Cohen).	Hatata has exaggerated his description adopting amplification strategies to dramatize and make the reader sympathize with the heroine as she is a victim. As for the translation of Ahlam Mosteghanemi's novel is concerned, the translator has highlighted the male presence by employing some terms denoting masculinity, in addition to the omission of some words and phrases used by the author in the novel that has feminist connotations
Jing-Jing (2019)	Translator’ ideology	Not explicitly mentioned	English -Chinese	*The Old Man and the Sea* by Ernest Hemingway (translator: Aileen Chang).	During the translation of The Old Man and the Sea, Eileen Chang consciously tries hard to preserve the meaning and style of the original text, while her strong feminist tendency leads her to unconsciously utter women's voices to a certain degree, not very explicitly, but implicitly.
Wang, Yu & Chen (2019)	Translator’ ideology	Not explicitly mentioned	Chinese-English	*Shuihu Zhuan* by Shi Naian (translator: Sidney Shapiro).	Sidney Shapiro has mitigated many of the stereotypes against women in patriarchal society present in the ST
Hou et al. (2020)	Feminist translation strategies.	Qualitative	English-Chinese	*Emma* by Jane Austen (translators: Sun Zhili and Zhu Qingying).	The female translator tends to use more feminist translation strategies in her practices consciously or unconsciously than the male translator
Li (2020)	Translators' subjectivity + gender consciousness	Not explicitly mentioned	English-Chinese	*The Color Purple* by Alice Walker (translators: Yang Renjing, and Tao Tie).	The male translator has paid no special attention to gender, whereas, the female translator has stronger feminist consciousness than the male translator who has shown deep-rooted patriarchal consciousness and a sense of gender discrimination in his translation, the female translator has shown feminist thought.
Jinga, Lihua (2020)	feminist translation strategies	Not explicitly mentioned	English-Chinese	*Pride and Prejudice* by Jane Austen (translators: Lei Limei and Sun Zhili).	The female translator makes the figures in the translation more vivid and artistic and conveys certain feminist ideas. So we can see that the feminist consciousness of the translator has a certain influence on the translation process.
Meng (2020)	Re-construction of gender in the English translation	Qualitative	Chinese-English	*Leaden Wings* by Zhang Jie (translator: Gladys Yang).	Textual and paratextual analyses show a paradox—feminist discourse and linguistic sexism—resides in the translation. Whilst feminist discourse is evident in both the paratexts, i.e. the preface, afterword and list of characters, and the text, the sexism embedded in the English language, represented in the translation by the use of male generic terms man/men and the female child term girl/girls to refer to adult women, finds its way into the translation.
Shaheen et al. (2021)	The discursive construction of feminist identities + the influence of the ideological position of the translator on the translation activity	Qualitative	Urdu- English	*The Dancing Girls of Lahore* by Louise Brown (translator: Dr. Naeem Tariq).	The position of the translator is reflected in the grammatical and lexical choices of the translation activity.
Mingli (2021)	Influence of ideological position of the translators in the translation activity	Qualitative	English- Chinese	*To the Lighthouse* by Virginia Woolf (translators: Qu Shijing and Ma Ainong).	Following the mainstream patriarchal ideology on women's role in society, Qu has presented Mrs. Ramsay as a perfectly idealized Victorian woman, which resonated with the social hierarchical rules for Chinese women at the time. Whereas, Ma has translated under a feminist socio-political scenario when the women's liberation movement was on the rise in China. She is perceived to create her translated text influenced by gender consciousness but She has expressed their feminist faith and concepts implicitly between lines.

**Table 3:** The miscellaneous findings of the studies applied feminist translation theory.

Author/s and year	Focus	Research Methodology	SL-TL	novel/s author-translators	Findings
Jinhua Li (2007)	Transnational film adaptation	Not explicitly mentioned	Chinese- English	*Brief einer unbekannten* by Stefan Zweig.	Adaptation as translation approach is a valuable TF for feminist cultural studies off Eastern-western dynamics
Yan Li (2010)	Representation of female body in TT/s	Not explicitly mentioned	Chinese- English + Chinese	*Jinsuo ji* by Eileen Chang -Self-translation.	The female body, as constructed in these four versions of the story, illustrates at once imprisonment by and a resistance to Chinese and Western aesthetic ideals.
Wu (2013)	Film adaptations	Not explicitly mentioned	English	Ang Lee's Sense and Sensibility by Jane Austen (translators: Ang Lee and John Alexander).	Males have dominated linguistic expression and translational norms
Ke Feng (2010)	Comparison between oriental feminism and western mainstream liberal feminism	Not explicitly mentioned	Chinese- English	*The Lost Daughter Of Happiness* (Fusang) by Geling Yan (translator: Cathy Silber).	This essay does not simply reflect a gender-based translation study but also addresses a focus on the cultural hegemony that has been imposed in the TT.
Mei (2019)	The role of dress—its production, materiality, and history—in the context of two English-language translations of the novel.	Not explicitly mentioned	Haitian-English	*La danse sur le volcan* (1957) by Marie Chauvet.	Transnational feminist literary translation praxis necessitates a sustained engagement with the material and imagined lives of objects in the longue durée, particularly fashion items such as scarves and dresses.

### 4.1 Impact of the gender consciousness and ideology of the translator on the translated activity

Most of the studies conducted concerning feminism and the literary translation of novels highlight that the gender consciousness and ideological positions of translators make an impact on the translation process (see Table 2). Feminist consciousness is the awareness of women that they belong to the dominated group, and this condition is not natural and socially determined and thus, women need to join together to change the situation, where they can enjoy independence like men (Lerner, 1993). The concept of ideology in translation is linked with a cultural turn in translation that emerged in the 1990s, and can be attributed to the works of Bassnett and Lefevere (1990), Lefevere (1992), and Tymoczko (2003). Translation is not done in a vacuum, but it is a form of rewriting which is influenced by certain ideological poetics and linguistic factors (Lefevere 1992).

Wang, Yu & Chen (2019) analysed the work of Shapiro who had translated one of the Chinese classic novels, *Shuihu Zhuan*, into English. The translation indicated how the gender consciousness had worked in the process of translation as the translator had mitigated some stereotypes against women present in the ST. This is an example of the translation of one translator, but most of the time, the selected research works are comparative (comparison of translations of male and female translators) mostly conducted in qualitative paradigms. Hsing (2011) worked on the Chinese translations of children's literature written in English by taking the writings of the number of male authors/translators and female authors/translators and had examined how gender differences of the ST and TT authors and translators respectively influenced the translation process. In this study, Hsing concluded that the female translators had intervened in the cultural references, and had produced their own identities. Conversely, male writers and translators had shaped the perspectives at the expense of women by using the language that was stronger and more aggressive, and had silenced women's voices, and thus made them invisible. The analysis depicted the arguments given by Spender (1980): “women are forced to see their experience and to justify male power from a male perspective from language that has been fashioned and controlled by men” (164). In the same ways, Li (2020) investigated gender influence on translation in the selected Chinese translation of *The Color Purple* by Alice Walker by taking the two translations of male and female translators and concluded that the male translators had paid no significant attention to gender, whereas female translators had stronger feminist consciousness by better conveying the inner feelings of the females.

In addition, gender differences in the translations of female alienation in the text have also been investigated. Tang (2018) by taking English to Chinese translations of male and female translators concluded that female translators had amplified women's awakening while keeping in view the feminist thoughts, and had chosen the word similar to the source meaning, whereas, male translators had omitted the expressions in ST indicating women's awakening. It indicated two things: either they had not understood women's awakening or they had deliberately ignored feminist thoughts. Thus, it had concluded that a “male translator's attitude towards women's awakening indicates their androcentric perspective that results from their gender conditioning influenced by the Chinese patriarchal culture and lack of knowledge from feminism” (178). Jinga and Lihua (2020) analyzed the English-Chinese translation of *Pride and Prejudice* by a female (Li Limei) and male translator (Sun Zhili), and concluded that the female translator had indicated more feminist consciousness in the translation activity. Furthermore, there are some studies, which have simply analysed how translations of male translators differ from female translators in terms of linguistic choices. Li and Zhang (2019) selected English to Chinese translations of *Persuasion* by Jane Austen, and concluded that the female translator had used more exclamatory sentences, rhetorical questions, reduplicated words and phrases, footnotes and final particles than the male translator. Mostly, the studies have highlighted the differences in translation between male and female translators but there is one piece of research found which has highlighted the similarities in their translations. Moghaddas (2013) had taken English to Persian translations of novels and had investigated the influence of translator's gender in the translation accuracy to analyse whether there was a major difference between the translations of male and female translators, and found no significant difference between both of the translations regarding translation accuracy. These studies have investigated the impact of gender consciousness in translated novels.

Moreover, the impact of the translator's ideology on the translation activity has also been one of the main and significant areas of research found in the studies on feminism and literary translations of novels. For example, Shaheen et al. (2021) investigated the ideological position of the translator in the Urdu translation of Brown's *The dancing girls of Lahore* done by Dr. Naeem Tariq and found that the ideological position of the male translator was reflected in the grammatical and lexical choices of the translation activity, and thus, had exposed the patriarchal structure of Pakistani society. This is the analysis of one translation of the selected novel. However, most of the studies investigating the impact of gender ideology on the translation process are comparative ones, and these have found that most of the time female translators have exhibited feminist ideologies and male translators have non-feminist ideologies. For example, Mingli (2021) investigated the influence of the ideological position of the translators in the English to Chinese translation of *To the Lighthouse* done by one male translator (Qu Shijing) and one female translator (Ma Ainong), and concluded that Qu had presented the female character as a perfectly idealized Victorian woman, which resonated with the social hierarchical rules for Chinese women at the time. However, the female translator (Ma) had translated under a feminist socio-political perspective when the women's liberation movement was on the rise in China, and thus, had expressed the feminist faith and concepts implicitly between lines. Furthermore, Modrea (2005) analysed the Spanish to English and the Spanish to French translations of *Tres Tristes Tigres* by Guillermo Cabrera Infante by looking at how the translators' ideologies were expressed in these translations and reviewed the reflection of feminist ideology in the translations of Levine, and few modification and direct translation in Bensoussan's translation. In the same way, in the English to Persian translation of Virginia Woolf's *Mrs. Dalloway* (1925), Mohammadi (2014) concluded that the male translator (Darush) had mostly used manipulative words and negative choices while the female translator (Taheri) had used positive choices.

Some studies have also found that the translator's identity as feminist influences the process of translation of the translator's sex or gender identity. For instance, Baya (2019) had taken two Arabic works: *Women at Zero Point* by Nawel Saadaouis and *Memory of the Flesh* by Ahlem Mosteghanemi with their English translations done by Hatata (male) and Raphael Cohen (male) respectively. The analysis of the first translation by Hatata had shown that there were feminist thoughts in the translation as he had adopted linguistic strategies to center the female heroine's perspective. Conversely, Raphael Cohen had emphasized the male presence by employing mostly terms that connoted masculinity.

#### 4.1.1 Feminist translation strategies in the translated novels

Another significant area investigated is the analysis of feminist translation strategies in the selected translated novels (see Table 2). Mostly, the studies have compared differences between the male and female usage of these strategies, and generally, the research works are analysed qualitatively with few using quantitative research. Firstly, those studies have been analysed, which have taken only one translation of the female translator (which are not comparative ones). Shou and Min (2017) took English-Chinese translations of Washington Irving's *The Legend of the Sleepy Hollow* and Hemingway's *The Old Man and the Sea,* and noticed that the translator Zhang Ailing had used the translation strategies of preface, supplementing and hijacking. These categories of translation strategies are attributed to Flotow (1991) where she explained in detail how feminist translators use them in the translation to show the female active presence. Feminist translators use prefaces in the process of womanhandling the text. The strategy of hijacking is linked to the linguistic manipulation performed by feminist translators used when the original text does not align with feminism. The strategy of hijacking is linked to the manipulation by feminist translators in which they use views that they do not link with feminism. These translation strategies are investigated in several studies about feminist translation. For instance, Shou and Min (2017) also analysed the usage of these feminist translation strategies in Chinese -English self-translation of Eileen Chang's The *Golden Cangue* and found a number of feminist translation strategies described above: prefaces, footnoting, supplementing, and hijacking. Jing – Jing (2019) also found the translation strategies of prefaces and hijacking by analysing English to Chinese translation of Hemingway's The Old Man and the Sea done by Ailen Chang. In the same way, Chen and Chen (2016) found the translation strategies of supplementing, prefaces and hijacking. Furthermore, most of the studies presented that the comparative usage of feminist translation strategies by the male and female translators has been investigated. Hou, Sun and Li (2020) analysed English to Chinese translations of *Emma* by Jane Austen done by one male translator (Sun Zhili) and one female translator (Zhu Qingying), and found that the female translator has used more feminist translation strategies than the male translator. In the same way, Qing Qiu (2019) analysed the Chinese translations of the male translator (Qu Shiji) and female translator (Ma Ainong) of the English novel Virginia Woolf's *To the Lighthouse*, and concluded that the female translator was more sensitive than the male translator in perceiving the various behaviours and psychology of the translated characters, and had used more the feminist translation strategies of hijacking and supplementing.

The above mentioned studies have employed qualitative approach. There are few studies which have also analysed the feminist translation strategies quantitatively. Allam (2018) in the Arabic to English translation of Reem Bassiouney's *Professor Hanna* done by Liala Helmy investigated the presence of feminist translational attitude, and concluded that the translation was woman-handled to emphasise female characters by using the feminist translation strategies: 7.2% (prefacing, footnoting), 75.3% (supplementing), 17.5% (hijacking). Additionally, one corpus based study had also been done by Bateni et al. (2013) who prepared the corpus of Persian translations of the novels (*Pride and Prejudice, Mrs. Dalloway, Wuthering Heights, Unaccustomed Earth, A thousand Splendid suns and Tess of the d'Urbervilles*) done by both male and female translators. They found that the female translators in this sample had used more of the feminist translation strategies of footnotes and hijacking than the male translators. Moreover, there are some studies that have reviewed the transference of gendered language in the translated text, which will be discussed in the next section (see Table 2).

### 4.2 Transference of gendered language in the translated novels

Palezek (2010) analysed the Polish-English translation of Tokarczuk's *Dom dzienny domnocny* performed by Antonia Lloyd and criticized the English translation for omitting most of the Tokarczuk play with gendered language and her challenges to the patriarchal structure in the original Polish. Palezek concluded that, although English lacked grammatical gender, it did not lack the corresponding patriarchal constructions. Thus, she acknowledged that it was difficult to transfer gender-specific linguistic concepts from ST to TT, but it was not impossible. Similarly, Miletch (2012) selected Spanish to English translation (done by the researcher himself) of a Venezuelan novel by Isaac Chocrón entitled in Spanish *Pronombres Personales*. The discussion of his analysis revolved around his own translation interventions as to differences in grammatical gender between the two languages. He stated that the novel had various challenges for the translator from the perspective of gender marking, and that this usually went unnoticed when translated from the traditional point of view. He admitted that he had intervened “as a translator in order to show issues regarding gender in the text” (1). The existence of texts that contained words that could be masculine or feminine in Spanish gave a challenge when transferring the Spanish text into English, a language that does not use grammatical gender, and with different gendered connotations than English. He added, “I did not translate the pronouns as they are usually translated but intervened to mark the particular gender of the pronouns (otherwise unnoticeable in many of the English pronouns such as *nosotros, nosotras* in Spanish, both -“we”- in English; *ellos, ella* in Spanish, both -“they”- in English) and to emphasize the gender issues present in the text that I feel combine the grammatical with the human. These are my most noticeable interventions in the text” (72).

The above discussion highlights that the conducted studies are mostly comparative (comparison of the work of male and female translators), and the research scholars have mostly analysed those translations of the novels which already read as feminist (*The Joy Luck, Pride and prejudice, Unaccustomed Earth* etc.) with a few non-feminist or actively misogynistic novels (*Shuihu Zhuan, The Old Man and the Sea*). They have highlighted that the gender consciousness and the ideological perspective of the translator leave an impact on the translation process, and that mostly the feminist translators have used those feminist translation strategies identified by Flotow (1991) and named above. Moreover, the issue of the transference of grammatical gender in TT is also seriously considered by feminist translators. Apart from these studies, there are also miscellaneous findings from the selected studies, which also highlight the ways feminist translation theory has been used in the analysis of translated novels (see Table 3).

The above-mentioned studies also mention that feminist translation theory has also been applied in the film adaptations, representations of the female body in TT/s, comparison between oriental feminism and western mainstream liberal feminism, among other findings. These are, however, beyond the scope of this piece of research.

## 5 Conclusion

In summary, this study presented a preliminary systematic review of the intersectionality between feminism and the translation of novels. This is significant research for those who work within the paradigm of feminist translation and it serves as an introduction to understanding the research perspectives on feminism and translation of novels applied and investigated. Moreover, it provides an understanding of the research contexts (languages, novels, investigated aspects) from the selected studies, and provides a solid rationale for further research on the matter. Based on the findings from the reviewed studies, the following needs for future research have emerged:

1. **Need to use corpus-based tools:** Currently, there are very few corpus-based research in this field. For instance, Bateni et al. (2013) have conducted a corpus-based study of the translated novels. This approach is common today for the systematic review of existing linguistics research. Thus, there is a need of conducting research using corpus analysis tools as they may uncover systematic linguistics patterns in the translated novels.
2. **Need to explore the translated works in other languages**: Most of the studies which analysed translated works involve English- Chinese translations and vice versa. There is a need to explore the translated works from other language families in different world regions from the perspective of feminist translation studies. Thus, the broader picture, regarding the issues under investigation (for instance, issues of translation from/into linguistic gendered language) can be brought to light in a variety of linguistic and cultural contexts.
3. **Need for conducting a comparison of Eastern/Western perspectives in translations:** Mostly, the research has been done with the comparison of male and female translations. There is a need to make a comparison of the feminist translations of western and eastern translators. Therefore, the difference in translation because of different cultural/perspectival positions should also be analysed. For instance, if the same work is done by the feminist translators belonging to Eastern and Western cultures, different perspectival positions may reflect on the same issue in their translated text.

## Declarations

### Author contribution statement

Musarat Yasmin: Conceived and designed the experiments; Analyzed and interpreted the data; Contributed reagents, materials, analysis tools or data.

Isra Irshad: Conceived and designed the experiments; Performed the experiments; Analyzed and interpreted the data; Wrote the paper.

### Funding statement

This research did not receive any specific grant from funding agencies in the public, commercial, or not-for-profit sectors.

### Data availability statement

No data was used for the research described in the article.

### Declaration of interests statement

The authors declare no conflict of interest.

### Additional information

No additional information is available for this paper.
